# Integrative analysis of ferroptosis in the hypoxic microenvironment of gastric cancer unveils the immune landscape and personalized therapeutic strategies

**DOI:** 10.3389/fonc.2024.1499580

**Published:** 2025-01-13

**Authors:** Xiao Xu, Liangling Fa, Xiaoxiao Sun, Fangfang Yang, Yongrui Liu, Jifu Song, Yongli Zhao, Jigang Dong

**Affiliations:** ^1^ Department of Radiation Oncology, Qingdao People’s Hospital Group (Jiaozhou), Jiaozhou Central Hospital of Qingdao, Qingdao, China; ^2^ Department of Pathology, Qingdao People’s Hospital Group (Jiaozhou), Jiaozhou Central Hospital of Qingdao, Qingdao, China; ^3^ Cancer Precision Medical Center, Qingdao University, Qingdao, China; ^4^ Department of Oncology, Linyi Cancer Hospital, Linyi, China; ^5^ Tianjin Medical University Cancer Institute & Hospital, National Clinical Research Center for Cancer, Tianjin’s Clinical Research Center for Cancer, Key Laboratory of Cancer Prevention and Therapy, Tianjin, China

**Keywords:** ferroptosis, immune landscape, immunotherapy, hypoxia microenvironment, tumor metabolism

## Abstract

**Background:**

Ferroptosis is a cell death mode caused by excessive accumulation of lipid peroxides caused by disturbance of intracellular metabolic pathway, which is closely related to iron and cholesterol metabolism homeostasis. Its regulation within the hypoxic metabolic tumor microenvironment (TME) has the potential to improve the effectiveness of tumor immunotherapy. The predictive role of ferroptosis in gastric cancer (GC) hypoxia TME, particularly in relation to TME immune cell infiltration, has not been fully explained.

**Methods:**

By analyzing the mRNA expression data of ferroptosis and hypoxia-related genes, a prediction model was constructed to evaluate further the predictive value of immune cell infiltration, clinical characteristics, and immunotherapy efficacy of gastric cancer, and the essential genes were validated.

**Results:**

Two distinct molecular states of ferroptosis-hypoxia were identified in GC. Notably, patients with high ferroptosis-hypoxia risk scores (FHRS) displayed significant levels of hypoxia and epithelial-mesenchymal transition (EMT), which were associated with unfavorable prognosis, increased chemoresistance, and heightened immunosuppression.

**Conclusions:**

This study demonstrates that ferroptosis under hypoxic conditions significantly affects the modulation of the tumor immune microenvironment. The FHRS can independently predict prognosis in gastric cancer. Assessing the molecular status of ferroptosis-hypoxia in individual patients will help in selecting more suitable immunotherapy regimens by providing a better understanding of TME characteristics and predicting immunotherapeutic outcomes.

## 1 Introduction

The latest global cancer statistics report highlights gastric cancer as one of the top five prevalent malignancies worldwide. The far-reaching public health impact of gastric cancer underscores the urgent need for an in-depth study of molecular biological mechanisms and improved treatment outcomes (1). Although chemotherapy and molecular targeted therapy have effectively prolonged the survival of gastric cancer patients, drug resistance remains a significant challenge. The molecular mechanism of drug resistance in gastric cancer remains incompletely understood, resulting in a lack of effective prevention and intervention in clinical practice (2). The advent of immunotherapy has led to a notable improvement in the survival rate of patients with advanced gastric cancer, challenging the dominance of chemotherapy and targeted therapy. Nevertheless, the efficacy of immune checkpoint blockade (ICB) therapy is constrained by the complicated tumor microenvironment and the inactivation of the immune system, which results in disparate outcomes. Consequently, there is a pressing necessity to develop more precise markers to assess the malignant process and forecast treatment response. Identifying these markers will facilitate the development of personalized therapeutic approaches, thereby enhancing patient outcomes and survival rates.

The concept of ferroptosis was initially developed in the context of tumor research (3). Researchers discovered this particular form of cell death, searching for a method to selectively induce death in cancer cells carrying RAS mutations. Recent evidence indicates that drug-induced ferroptosis can reverse drug resistance, a crucial tumor suppressor mechanism (4). In addition, ferroptosis enhances the infiltration and activity of tumor immune cells and decreases the recruitment and function of immunosuppressive cells, thereby reducing immunosuppression and promoting tumor immunosurveillance and immune-mediated tumor clearance. The study by Li Y et al. proposes a novel strategy to enhance immunogenic cell death (ICD) and the cascade effect of T-cell activity through ferroptosis for effective tumor therapy (5, 6). Although the mechanisms of interaction between ferroptosis and ICB therapy are still under investigation, current evidence suggests that the facilitating role of ferroptosis may provide a new strategy for enhancing the response to ICBs in certain refractory tumors. Further studies are required to elucidate the specific mechanisms of these interactions and to validate the efficacy of the combination strategy of ferroptosis inducers and ICB therapy in clinical trials.

Hypoxia profoundly affects tumor metabolism and microenvironment, including angiogenesis, cell proliferation, invasion, and metastasis. These processes reduce apoptosis, differentiation, and ferroptosis, thereby promoting tumor immunosuppression and escape (7–9). Ameliorating hypoxia can reshape the immunosuppressive tumor microenvironment by reducing the intratumoral invasion of M2-type tumor-associated macrophages and decreasing PD-L1 expression in tumor cells (10). Furthermore, hypoxia can induce EMT in cancer cells, which promotes the stem-like features of cancer cells and leads to tumor therapy resistance. In human cancer cell lines and organoids, a highly mesenchymal state unequivocally implies a selective susceptibility associated with ferroptosis (3, 11). The central molecule in the cellular response to hypoxia is the hypoxia-inducible factor (HIF). The HIF signaling pathway senses metabolic changes due to cellular hypoxia, regulates cell proliferation, and induces inflammatory responses (12). It was found that HIF-1α upregulates SLC1A1 to enhance glutamate-cystine transport efficiency, thereby driving solid tumor resistance to ferroptosis (13). Additionally, LDHA-activated lactate accumulation is promoted by HIF-1α to enhance ferroptosis resistance (14, 15). Another researcher prepared nanoparticles (CI@HSA NPs) encapsulating capsaicin (CAP) and the photosensitizer IR780 to enhance the efficacy of photodynamic therapy (PDT) on osteosarcoma. The nanoparticles were designed to release capsaicin, which has been demonstrated to promote osteosarcoma ferroptosis and improve the hypoxic microenvironment (16). Combining HIF-1α inhibitors with ferroptosis inducers represents a novel strategy for solid tumor therapy. These findings elucidate the molecular mechanism of hypoxia-induced ferroptosis resistance in solid tumors and provide new theories and strategies for solid tumor treatment.

This study integrated genomic and clinical data from gastric cancer samples from four datasets to identify and comprehensively evaluate two ferroptosis clusters. Additionally, two hypoxia molecular subtypes were identified using the same method. Both molecular types were closely associated with the prognosis and immune cell infiltration signaling pathway in gastric cancer patients, suggesting that both ferroptosis and hypoxia play an integral role in shaping the specific characteristics of the individual tumor microenvironment. Consequently, these molecular subtypes were combined into a two-dimensional index, designated the ferroptosis-hypoxia subtypes. Further analysis demonstrated that the ferroptosis-hypoxia subtypes were closely associated with prognosis, tumor immune cell infiltration, and mesenchymal characteristics of gastric cancer patients. Based on these findings, we developed a scoring system that quantifies the ferroptosis-hypoxia status of individual patients. The scoring system enables the selection of individualized treatment regimens for patients and the optimization of treatment strategies by assessing the ferroptosis-hypoxia status.

## 2 Materials and methods

### 2.1 Data preparation

The gastric cancer gene expression data and clinical annotations were acquired from The Cancer Genome Atlas (TCGA) database, which is publicly accessible at https://portal.gdc.cancer.gov/repository. Validation cohort data from GSE112302, GSE84437, and ACRG/GSE62254 were downloaded from the Gene Expression Omnibus (GEO, https://www.ncbi.nlm.nih.gov/geo) database. TCGA-STAD copy number variation (CNV) data were also extracted from the UCSC Xena database (https://xena.ucsc.edu/). The anti-pd-1 treatment cohort PRJEB25780 data was obtained from the Tumor Immune Dysfunction and Exclusion Database (TIDE, http://tide.dfci.harvard.edu/) (17). The clinical information, including microsatellite instability (MSI) status, remission, and Lauren typing, was extracted from the manuscript of Mayakonda et al. (18). A total of 1,121 GC patient samples were included in this study.

The “ComBat” function provided by the R package “sva” removed the batch effect. “ComBat” is a classical Bayesian-based analysis that utilizes known batch information for the correction of high-throughput data, ensuring that the comparisons across datasets are accurate and meaningful (19, 20).

Ferroptosis-related genes (FRGs) were obtained from the FerrDb website (http://www.zhounan.org/ferrdb), which is the first database of ferroptosis regulatory factors, biomarkers, and ferroptosis disease associations (21). We removed duplicate genes and obtained 380 FRGs for subsequent analyses (**Supplementary Table 1**). Hypoxia-related genes (HRGs) were obtained from the Molecular Signatures Database (MSigDB, https://www.gsea-msigdb.org/gsea/msigdb) (22). HIF-1 pathway target genes were downloaded from the Kyoto Encyclopedia of Genes and Genomes database (KEGG, https://www.kegg.jp/; ID:map04066), including genes associated with “increasing oxygen delivery” and “decreasing oxygen consumption”. The negative and positive regulator genes of ferroptosis were downloaded from the Gene Ontology database (GO, https://geneontology.org/; GO: 0160020, GO: 0110076).

All data in TCGA, GEO, TIDE, FerrDb, KEGG, GO, and MSigDB are publicly available and adhere to the data access and release policies of the respective databases.

### 2.2 Detection of ferroptosis molecular subtypes and hypoxia molecular subtypes

The FPKM values of the RNA sequencing data from the TCGA-STAD dataset were transformed into TPM using the R package “TCGAbiolinks” (23). The differentially expressed FRGs and HRGs (FDR<0.01, |logFC|>1) were analyzed and screened from gastric cancer and para-carcinoma samples utilizing the R package “limma” (24). Unsupervised cluster analysis was performed with the “ConsensusClusterPlus” package to identify ferroptosis and hypoxia molecular subtypes based on the mRNA expression profiles of the differentially expressed genes (DEGs) (25). A consensus clustering algorithm was used to determine cluster number and stability. The analysis was repeated 1000 times to ensure classification stability. Subsequently, patients from the TCGA-STAD, GSE84437, GSE62254, GSE112302, and PRJEB25780 cohorts were categorized for subsequent analysis.

### 2.3 Tumor microenvironment characterization and functional enrichment analysis

To further enhance comprehension of how ferroptosis impacts the tumor immunological microenvironment, we applied the CIBERSORT analysis (http://cibersort.stanford.edu/) (26). The publication of CIBERSORT was released in the scientific journal Nature Methods in 2015. It is the most commonly referenced instrument for estimating and analyzing the infiltration of immune cells. We utilized CIBERSORT computations to conduct analyses on immune cell infiltration to identify the properties of the immunological microenvironment.

The ESTIMATE algorithm, which estimates the level of infiltrating stromal and immune cells in malignant tumor tissues, employs expression data to generate scores. These scores are used to calculate the level of infiltrating stromal cells and immune cells, as well as tumor purity.

GO and KEGG analyses were conducted on DEGs (FDR<0.05) using the “clusterProfiler” R package (27). To investigate the biological processes, we downloaded the gene set “c2.cp.kegg. v7.4” from the MSigDB database to perform Gene Set Variation Analysis (GSVA) enrichment analysis. GSVA is an unsupervised approach for quantifying alterations in biological pathways and processes within expression dataset samples. A collection of signaling pathways was examined to investigate the matrix state within the tumor microenvironment, including TGF-EMT, MAPK, NOTCH, KRAS, HALLMARK HYPOXIA, and HIF-1 (28). Subsequently, we employed single-sample Gene Set Enrichment Analysis (ssGSEA) to investigate the mechanisms underlying the generation of TME features (29). We acquired a collection of genes associated with EMT markers from Mariathasan et al., including EMT1, EMT2, EMT3, angiogenic signature, TGF-β response signature of pan-fibroblasts (Pan-FTBRS), and WNT targets (30).

### 2.4 Identification of characteristic molecular subtypes of ferroptosis-hypoxia

We proceeded to combine the above ferroptosis and hypoxia status into a two-dimensional index. The patients were classified into three groups: the ferroptosis-hypoxia (F-H) molecular subtypes A, B, and Mix. Patients belonging to both the ferroptosis cluster A and the hypoxia cluster A were identified as F-H subtype A, patients belonging to both the ferroptosis cluster B and the hypoxia cluster B were classified as F-H subtype B, and the remaining patients were classified as F-H subtype Mix. The F-H subtype expression profiles in groups A and B were compared to identify DEGs(FDR<0.001, |log FC|>1). Unsupervised clustering analysis was performed on the DEGs mentioned above to construct gene subtypes related to the F-H molecular subtypes.

The DEGs were overlaid with FRGs and HRGs and subjected to univariate Cox analysis. The ferroptosis-hypoxia-related prognostic DEGs were identified for further analysis (*p*<0.05). In order to reduce overfitting, we constructed a prognostic model utilizing Lasso-penalized Cox regression analysis (31). The Lasso algorithm was employed for variable selection and shrinkage, and the R package “glmnet” was utilized to filter out variables with less information. The model was constructed using the TCGA dataset as the training set and the GSE62254 dataset as the independent test set to obtain the optimal combination of variables. The regression model used the normalized expression matrix of candidate prognostic DEGs as independent factors. Meanwhile, OS and patient status in the TCGA were considered response variables. The penalty parameter (λ) of the model was chosen using tenfold cross-validation according to the minimal criteria, which corresponds to the value of λ that minimizes the partial likelihood deviation. Risk scores for patients were computed by utilizing the normalized expression levels of each gene and their related regression coefficients. The formula is as follows: score = e^sum (each gene’s expression × its corresponding coefficient)^. Subsequently, patients were divided into high-risk and low-risk groups based on the median risk score. Time-dependent ROC curve analysis of subjects at 1, 3, and 5 years was performed using the “timeROC” R package to assess the predictive power of the gene signature. Additionally, the AUC values of survival ROC curves were calculated to assess the performance of prognostic prediction models. Regression models were constructed by integrating risk scores and other clinical factors. Nomogram plots were employed to visually represent the relationship between variables in the prediction model. These plots were displayed on the same plane and at a specific scale. Prognostic calibration plots were used to analyze the fit of the model to the actual situation, with the objective of testing the consistency of the nomogram survival probability prediction with the actual observation.

### 2.5 Assessment of the correlation between clinical characteristics and predictive risk scores

The Kruskal-Wallis test assessed the disparities between clinical characteristics and risk scores generated by multiple data analyses. Spearman correlation analysis was employed to determine the correlation between risk scores. Furthermore, a Kaplan-Meier survival analysis (log-rank test) was conducted to evaluate the impact of patients on overall survival.

### 2.6 Analysis of the association between somatic mutations and risk scores

The WES data, obtained from the TCGA portal and analyzed using VarScan2, included single nucleotide variants (SNVs), insertions (INS), single nucleotide polymorphisms (SNPs), and deletions (DEL). The somatic mutation data were displayed employing the “maftools” software package, which is capable of processing Mutation Annotation Format (MAF) files (18). The calculation method for the tumor mutation burden (TMB) of each patient is as follows: the total number of variants was divided by the total exon length.

### 2.7 Therapeutic strategies based on ferroptosis-hypoxia risk scores

Jiang et al. established the Tumor Immune Dysfunction and Exclusion (TIDE) to mimic tumor immune escape mechanisms, including T-cell dysfunction and T-cell rejection (32). Consequently, TIDE can be employed to forecast the efficacy of immunotherapy. A higher TIDE score reflects an immune escape phenotype in the tumor and a poorer response to ICBs.

The Immunophenoscore (IPS) was obtained from The Cancer Immunome Atlas (TCIA, https://tcia.at/home). As a molecular marker of the immune response, the IPS provides an excellent indication of the immune landscape within the tumor. It was determined that a scoring scheme could be created by identifying genes related to the immune system. A higher IPS score is indicative of higher immunogenicity levels. In this context, IPS can be employed to assess immunotherapy efficacy in GC patients.

The Genomics of Drug Sensitivity in Cancer (GDSC) database was utilized to evaluate the susceptibility of ferroptosis-hypoxia states to chemotherapeutic drugs. The IC_50_ is calculated based on the “pRRophetic” software and represents the concentration at which the inhibitory effect reaches half the maximum value (33, 34).

### 2.8 Pan-cancer analysis

We further systematically summarized the clinical relevance and immunological characteristics of risk scores in pan-cancer to externally validate the general applicability of risk scores. Gene expression and associated clinical information for 33 tumors were downloaded from the TCGA database.

### 2.9 Quality control and standardization of scRNA-seq data

Download the six samples of the GSE112302 dataset from the GEO website, which includes scRNA-seq data from 402 GC cells. Then, create a “Seurat” object containing basic information about the single-cell dataset using the “CreateSeuratObject” function in the “Seurat” R package. Subsequently, data quality control was conducted. The scRNA-seq data underwent normalization applying the “LogNormalize” method, and a variance analysis was performed to identify the top 1500 genes with highly variable characteristics. Subsequently, the dimensionality of the data was reduced through the application of principal component analysis (PCA). The dimensions exhibiting significant separation were subjected to PCA at a false discovery rate (FDR) of less than 0.05, and the first 15 principal components (PCs) were subsequently downscaled by the t-distributed stochastic neighbor embedding (tSNE) algorithm to yield principal component clusters. The marker genes in each cluster were identified using the criteria of log2 [fold change (FC)]>0.5 and FDR<0.05. The clusters were annotated using the marker gene-based “Single” R package.

### 2.10 Immunohistochemical analysis of clinical validation cohort

A total of 30 surgical specimens of GC, along with 25 matched paracancerous tissues, were collected from Qingdao People’s Hospital Group (Jiaozhou) (hereafter referred to as our hospital). In order to evaluate the levels of expression of central genes (SDC2, RGS4, SERPINE1, DUSP1, and CAV1), immunohistochemistry (IHC) was conducted using GTVisionTM III Detection System. According to the instructions from the manufacturer, the following antibodies were used for immunohistochemical staining: 67088-1-Ig, 14530-1-AP, 66261-1-Ig, T56588S, and 16447-1-AP. Two pathologists, unaware of the patient’s clinical information, evaluated the immunohistochemical staining. In case of a discrepancy in the assessments, a third pathologist conducted an independent review. Ten optical fields were examined in each diseased region using a high-power lens (×400). The IHC staining score was used as the definitive criterion for judging the staining. The IHC staining score is calculated by multiplying the staining area score by the staining intensity score. The score for the staining area was assessed on a scale ranging from 0 to 4, with 0 representing a staining area of ≤10%, 1 representing a staining area of 11 to 25%, 2 representing a staining area of 26 to 50%, 3 representing a staining area of 51 to 75%, and 4 representing a staining area of >75%. The staining intensity score was categorized as 0: negative, 1: weak, 2: moderate, or 3: strong. The IHC staining scores were dichotomized as follows: scores below six were defined as low expression, while scores above six were defined as high expression.

### 2.11 Statistical analysis

A student t-test was employed to assess differential gene expression between tumor and adjacent non-tumor tissues. Comparisons between the two groups were conducted via the Wilcoxon rank-sum test. Additionally, multiple comparisons were performed by the Kruskal-Wallis test. Cut-off points for each subgroup were determined using the “survminer” R software package. The Kaplan-Meier method was employed to analyze overall survival (OS) between subgroups, with the log-rank test used to assess the significance of the results. The chi-squared test was utilized to compare proportion differences. Univariate and multivariate Cox regression analyses were performed to determine the independent factors that predict OS.

## 3 Results

### 3.1 Identification of molecularly characterized subtypes of ferroptosis and hypoxia in GC

**Figure 1:**
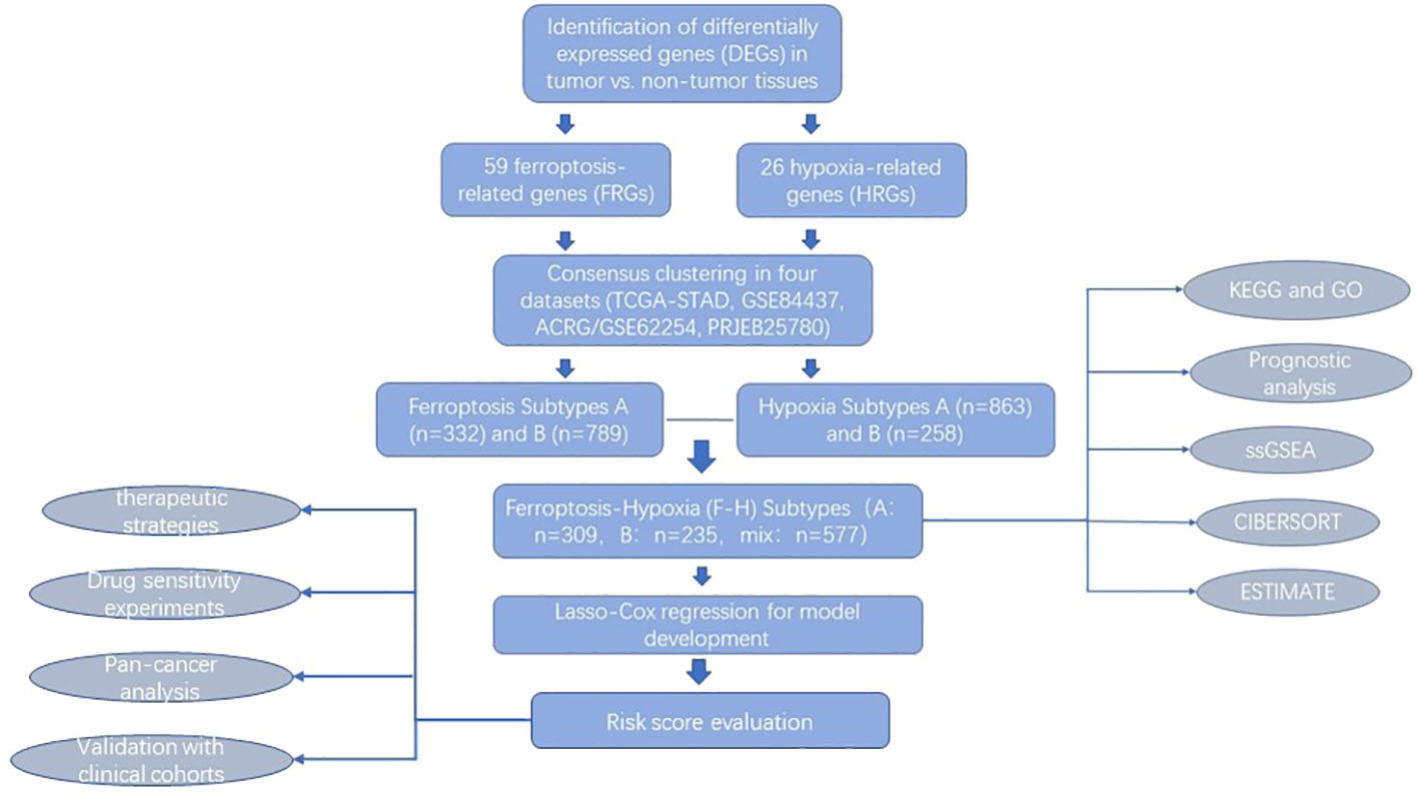
Flow chart of our study.

**Figure 2:**
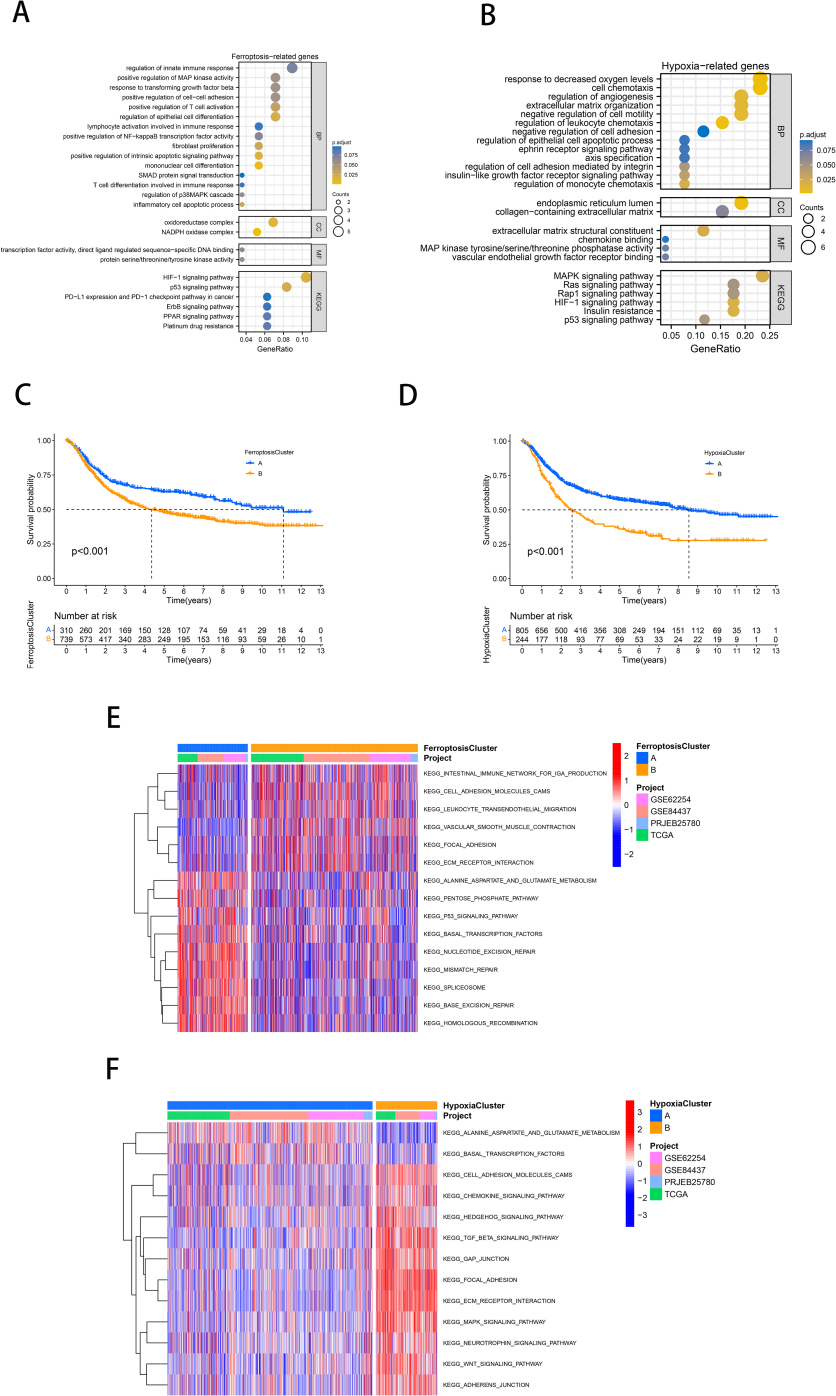
Identification of molecularly characterized subtypes of ferroptosis and hypoxia in GC. **(A, B)** GO and KEGG analysis based on FRGs **(A)** and HRGs **(B)**. **(C, D)** Kaplan-Meier curves of GC patients for ferroptosis molecular subtypes **(C)** and hypoxia molecular subtypes **(D)**. **(E, F)** GSVA analysis revealed distinct activations of biological pathways in ferroptosis clusters **(E)** and hypoxia clusters **(F)**. Blue represented the inhibition pathway, and red represented the activation pathway.

The flow chart shows our research procedure (**Figure 1**). In the TCGA-STAD cohort, we have found 59 FRGs that are expressed differently across tumor tissues and adjacent non-tumor tissues (FDR<0.001, |logFC|>1; **Supplementary Figure 1A**, **Supplementary Table 2**) using the “limma” R package. In KEGG and GO analysis, these DEGs were found to be enriched in several pathways, including positive regulation of MAP kinase (MAPK) activity, response to TGF-β, enhancement of cell-cell adhesion, and promotion of T-cell activation, activation of immune response, fibroblast proliferation, platinum resistance, apoptotic process of inflammatory cells, oxidoreductase complex, p53 signaling pathway, HIF-1 signaling pathway, and many other oncology-related pathways (**Figure 2A**). The same identification method was employed to screen for 26 HRGs that exhibited differential expression between gastric cancer tumors and normal tissues (FDR<0.001, |log FC|>1; **Supplementary Figure 1B**, **Supplementary Table 2**). These HRGs were enriched in many biological functions related to angiogenesis, modulation of extracellular matrix components, modulation of cell adhesion, chemotaxis of immune cells, and a variety of oncogenesis-related pathways (MAPK, Ras, Rap1, insulin-like growth factor receptor, p53, HIF-1) (**Figure 2B**).

It is reasonable to hypothesize that ferroptosis and hypoxia play a significant role in tumor progression. Subsequently, a consensus clustering approach was used to identify characteristic molecular subtypes of ferroptosis in gastric cancer. Based on the mRNA expression profiles of 59 FRGs, GC patients from 4 cohorts were clustered into two ferroptosis clusters, A and B (A: *n*=332, B: *n*=789; **Supplementary Figure 1C**). PCA confirmed that these two subtypes could be altogether distinguished (**Supplementary Figure 1D**). Prognostic analysis showed a significant survival advantage for ferroptosis cluster A compared to subtype B (*p*<0.001; **Figure 2C**). Following the same approach, we classified the gastric cancer samples from the four cohorts into two hypoxia molecular subtypes, A and B (A: *n*=863, B: *n*=258; **Supplementary Figure 1E**). PCA confirmed that these two subtypes could be fully distinguished (**Supplementary Figure 1F**). Similarly, prognostic analyses showed that hypoxia cluster A exhibited a notable survival benefit (*p*<0.001; **Figure 2D**).

As illustrated in **Figure 2E**, ferroptosis cluster A was significantly enriched in the pentose phosphate pathway, alanine aspartate and glutamate metabolism, DNA repair, and p53 signaling pathway. Subtype B was significantly enriched in the influence of the intestinal immune network for IgA production, cell adhesion molecules (CAMs), and Extracellular matrix and (ECM) receptor interaction. According to **Figure 2F**, hypoxia cluster B was significantly enriched in the stromal activation and oncogenic pathways (ECM receptor interaction, CAMs, TGF-β signaling pathway, MAPK signaling pathway, WNT signaling pathway).

### 3.2 Identification of molecular subtypes for combined ferroptosis-hypoxia

**Figure 3:**
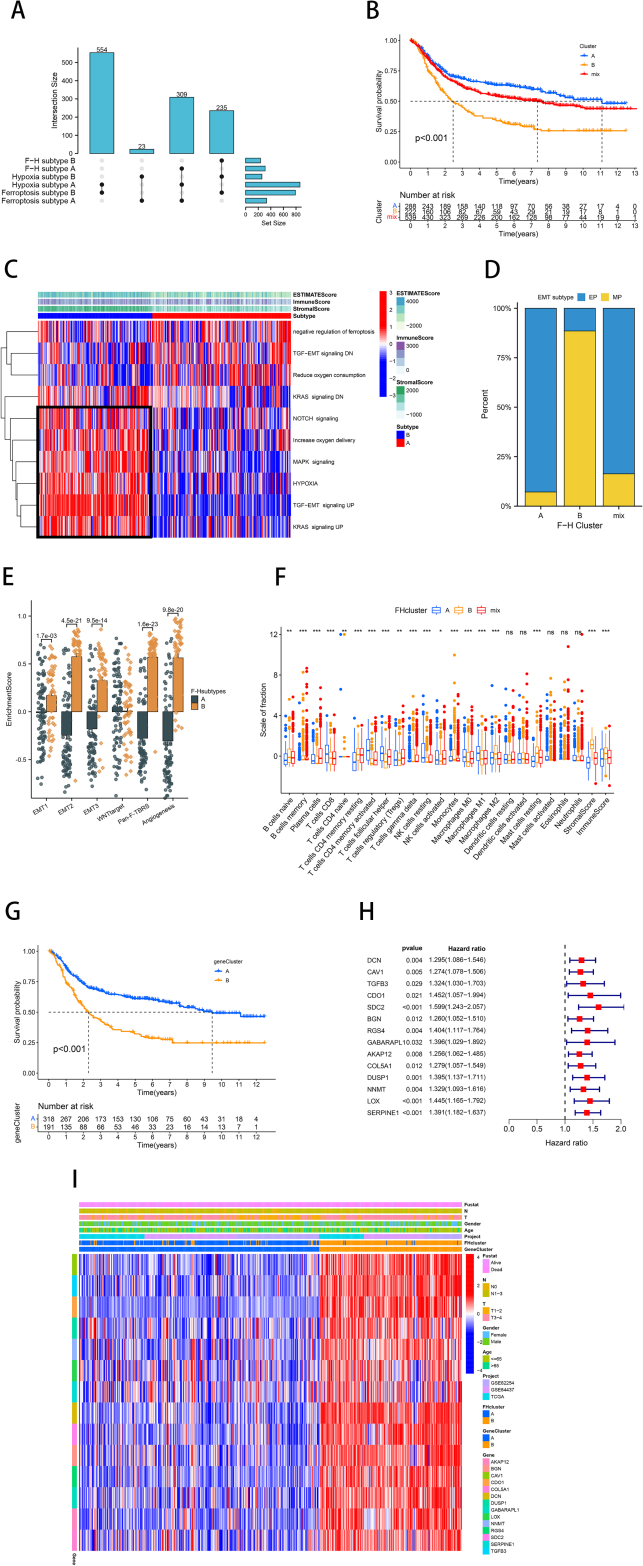
Identification of molecular subtypes for combined ferroptosis-hypoxia. **(A)** The upSet diagram shows the composition of ferroptosis-hypoxia subtypes. **(B)** Kaplan-Meier curves of GC patients for ferroptosis-hypoxia subtypes. **(C)** Heat map illustrating the relationship between ferroptosis-hypoxia subtypes, ESTIMATE score, and biological pathways. **(D)** Differences in ferroptosis-hypoxia subtypes for EMT typing. **(E)** Box plot showing ssGSEA analysis of EMT differences between ferroptosis-hypoxia subtypes. **(F)** Box plots of immune infiltration levels in ferroptosis-hypoxia subtypes. ns, not significant; *p<0.05; **p<0.01; ***p<0.001. **(G)** Kaplan-Meier curves for the gene clusters of GC patients. **(H)** Forest plots illustrating the Univariate Cox regression analysis of ferroptosis-hypoxia marker genes. **(I)** Heat map illustrating the relationship between ferroptosis-hypoxia subtypes associated gene expression, ferroptosis-hypoxia clusters, and various clinicopathological features.

The preceding analysis demonstrated that both the ferroptosis and hypoxia molecular subtypes exhibit favorable prognostic value and distinctive tumor-related biological characteristics. Therefore, based on the above ferroptosis and hypoxia status, we further combined them into a two-dimensional metric and categorized patients into ferroptosis-hypoxia (F-H) subtypes A, B, and mix (A: *n*=309, B: *n*=235, mix: *n*=577; **Figure 3A**). The survival analysis revealed that patients with F-H subtype A exhibited the highest survival rate, while those with F-H subtype B exhibited the poorest prognosis (*p*<0.001; **Figure 3B**).

The heatmap demonstrated the ESTIMATE score of F-H subtypes and the enrichment of multiple biological pathways (**Figure 3C**). Notably, we found that the NOTCH signaling pathway, MAPK signaling pathway, hypoxia, and activation of TGF-EMT signaling pathway were highly expressed in F-H subtype B, while TGF-EMT signaling down regulation were highly expressed in F-H subtype A. In 2018, Oh et al. conducted an analysis of genomic and proteomic data to distinguish between two separate categories of gastric cancer: mesenchymal phenotype (MP) and epithelial phenotype (EP) (35). These two subtypes showed markedly different survival and chemotherapy sensitivity. Based on their study, we found that the interstitial features of F-H subtype B were more prominent (**Figure 3D**). The EMT analysis was conducted using the single sample Gene Set Enrichment Analysis (ssGSEA) method, demonstrating significantly enhanced stromal activity in F-H subtype B. This was evidenced by the enrichment of EMT, pan-fibroblast TGF-β response signature (Pan-F-TBRS), and angiogenic pathways, which supported our hypothesis (**Figure 3E**). CIBERSORT analysis revealed a significant enrichment of immune-activated cells in F-H subtype A, including CD8+ T cells, M1 macrophages, and CD4+ T cells. The immunosuppressive cells, such as M2 macrophages, are abundant in subtype B (**Figure 3F**).

We further compared the expression profiles of F-H subtype A and F-H subtype B. A total of 520 DEGs were identified (FDR<0.001, |log FC|>1; **Supplementary Figure 1G**). In order to further verify this regulatory mechanism, an unsupervised clustering was performed on the basis of these DEGs. The patients were categorized into two F-H genomic patterns, referred to as gene clusters A and B (A: *n*=342; B: *n*=201). The prognosis for gene cluster A was demonstrably superior to that of gene cluster B (**Figure 3G**). We overlapped 520 DEGs with FRGs and HRGs to identify 25 ferroptosis-hypoxia (F-H) marker genes (**Supplementary Figure 1H**). Univariate Cox analysis was performed to select 14 F-H prognostic genes (*p*<0.05; **Figure 3H**). The heatmap demonstrated that the gene clusters were similar to the F-H molecular subtypes. Furthermore, the expression of the 14 prognostic DEGs was found to be significantly upregulated in gene cluster B and F-H subtype B (**Figure 3I**).

### 3.3 Construction and validation of a prognostic model based on ferroptosis-hypoxia genes

**Figure 4:**
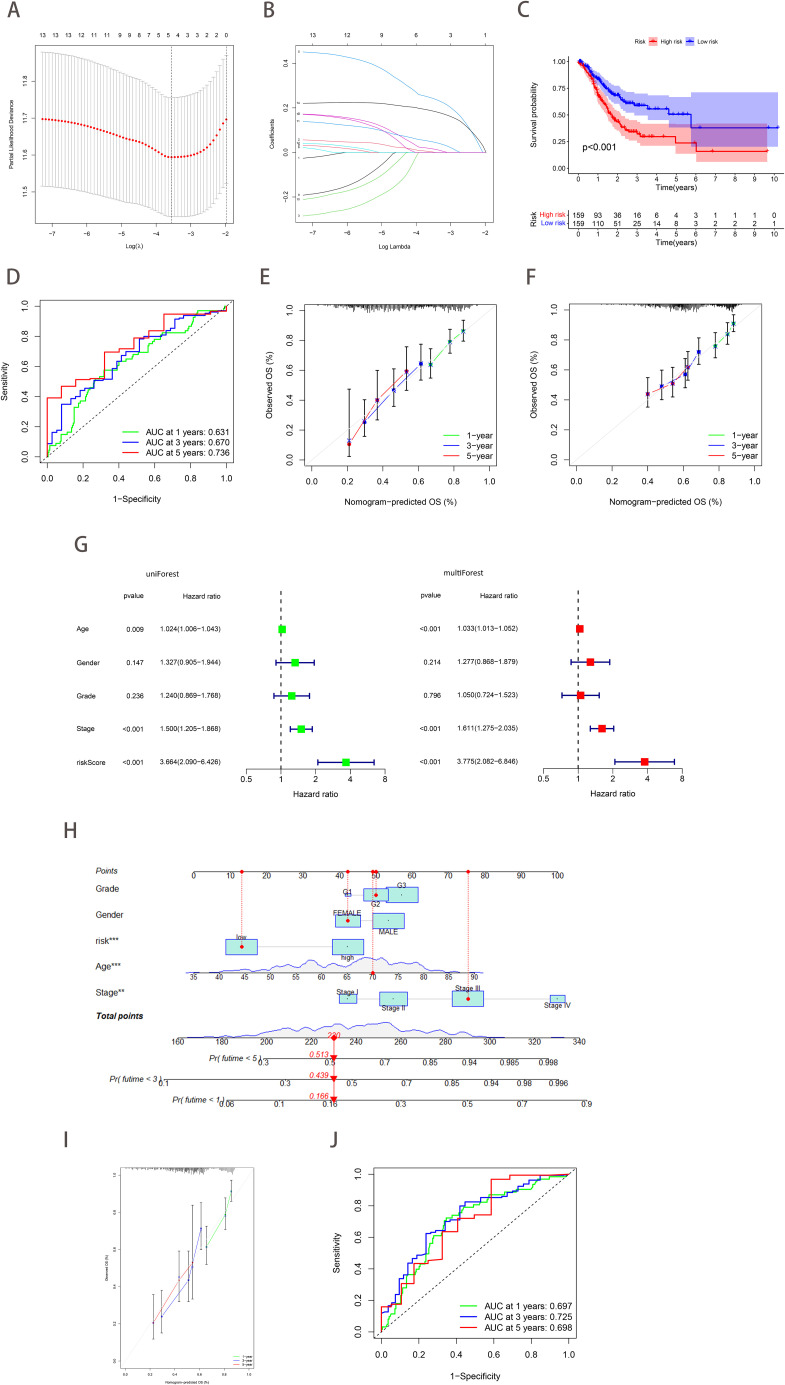
Construction and validation of a prognostic model based on ferroptosis-hypoxia genes. **(A)** The log value of the independent variable lambda (the abscissa represents the confidence interval of each lambda, and the ordinate represents errors in cross-validation). **(B)** The changing trajectory of each independent variable (the abscissa represents the corrected lambda, and the ordinate represents the coefficient of the independent variable). **(C)** The K-M curve of the five-gene signature-based stratification in TCGA training cohort. **(D)** The 1-, 3-, and 5-year ROC curve is based on five-gene signature stratification. **(E, F)** The calibration plot evaluates the fit analysis of the model to the actual situation in TCGA **(E)** and GSE62254 **(F)**. **(G)** Univariate and multivariate Cox analysis of risk score. **(H)** Nomogram plot of the prognostic multivariate regression model. **(I)** Prognostic Calibration plot evaluating the fit analysis of the model to the actual situation. **(J)** The 1-, 3-, and 5-year ROC curve is based on the prognostic multivariate regression model.

Considering the heterogeneity and complexity of individual gastric cancer patients, analyses based on patient groups alone are insufficient for accurately predicting the prognosis of individual GC patients. Therefore, we established a prognostic model by Lasso-Cox regression analysis using the expression profiles of the above 14 genes. A signature consisting of five genes was identified utilizing the optimum value of λ (**Figures 4A, B**). The risk score was computed employing the following methodology: e ^(0.0029 * expression level of CAV1 + 0.2445 * expression level of SDC2 + 0.0168 * expression level of RGS4 + 0.0485 * expression level of DUSP1 + 0.1873 * expression level of SERPINE1)^. Patients in TCGA (training cohort) were divided into high-risk (*n*=159) and low-risk (*n*=159) groups. Kaplan-Meier curves demonstrated that patients in the high-risk group exhibited a notably lower OS than patients in the low-risk group (**Figure 4C**). Time-dependent ROC curves further confirmed the excellent sensitivity and specificity of the risk score in predicting GC survival outcomes (1-year AUC=0.631, 3-year AUC=0.670, 5-year AUC=0.736; **Figure 4D**). The above conclusions were also validated in the GSE62254 cohort (**Supplementary Figures 2A, B**). The prognosis calibration chart was employed to assess the alignment between the model and the actual situation, with calibration curves for 1-, 3-, and 5-year OS analyzed. **Figures 4E, F** demonstrated that the risk score in the TCGA and GSE62254 cohorts had a predictive effect on the outcome of gastric cancer patients aligned with the actual situation. Subsequently, univariate and multivariate Cox analysis demonstrated the independent prognostic value of the risk score (*p*<0.001; **Figure 4G**). Nomograms were employed to visualize the risk score in conjunction with other clinical risk factors (grade, gender, age, stage) to construct a predictive multivariate regression model (**Figure 4H**). **Figures 4I, J** illustrated the calibration curves and time-dependent ROC curves for 1-year, 3-year, and 5-year OS. These figures demonstrate that the risk score has excellent predictive efficacy for the outcome of gastric cancer patients. This conclusion was similarly validated in the GSE62254 cohort (**Supplementary Figures 2C–H**).

### 3.4 The landscape of genetic variation of ferroptosis-hypoxia risk scores

**Figure 5:**
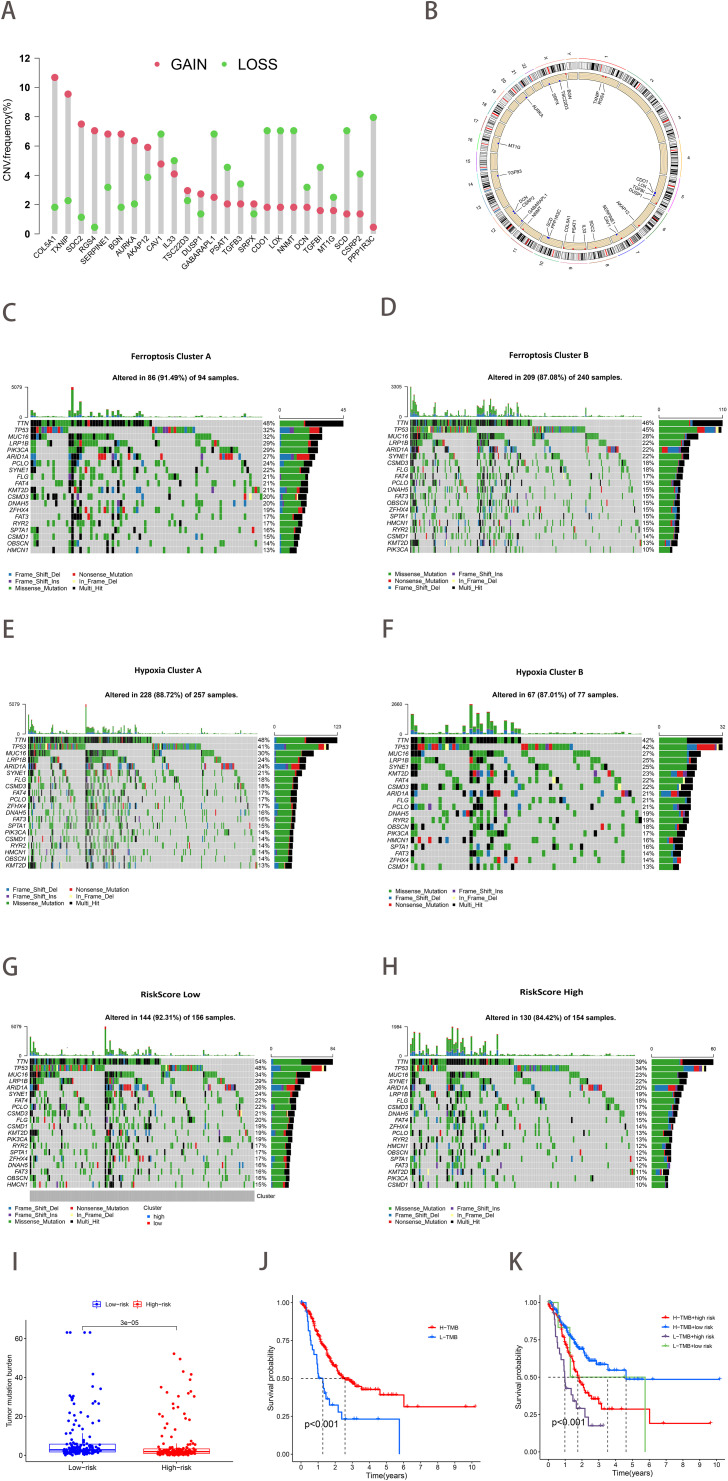
The landscape of genetic variation of ferroptosis-hypoxia risk scores. **(A)** The frequency of CNV variation of 25 ferroptosis-hypoxia genes. The height of the column represented alteration frequency. Green dots indicate deletions; red dots indicate amplifications. **(B)** CNV alteration locations for 25 ferroptosis-hypoxia genes. **(C–H)** Waterfall plots of tumor somatic mutations in patients with low ferroptosis subtypes A **(C)**, ferroptosis subtypes B **(D)**, hypoxia subtypes A **(E)**, hypoxia subtypes B **(F)**, low-risk group **(G)**, high-risk group **(H)**. Each column represents one patient. The top bar indicates the degree of tumor mutation. The numbers on the right indicate the frequency of mutations in each gene. The bars on the right show the proportion of different types of mutations. Stacked bar graphs show the conversion rate for each sample. **(I)** Boxplots illustrating the difference in tumor mutation burden between risk score groups. **(J)** Kaplan-Meier curve for tumor mutation burden groupings. **(K)** Kaplan-Meier curve for risk score and tumor mutation burden.

The study of copy number variation (CNV) demonstrated that CNV was prevalent among the 25 ferroptosis-hypoxia genes, with a significant number concentrated on copy number amplification (**Figure 5A**). **Figure 5B** depicts the chromosomal location of CNV in ferroptosis-hypoxia genes. The somatic mutation data indicated that both the ferroptosis cluster A and the hypoxia cluster A exhibited higher mutation frequencies than cluster B. Furthermore, when applying the risk score for grouping, the difference in mutation frequency between the low and high groups became more significant (**Figures 5C–H**). The quantification analysis of TMB demonstrated that, among patients with gastric cancer, those with a low-risk score exhibited a relatively higher TMB, and a higher TMB was associated with improved survival, which is consistent with our results (**Figures 5I–J**). In light of the possible synergistic impact of TMB and risk score on evaluating prognosis for patients, we proceed with performing stratified prognostic analysis. A significant survival advantage was observed in patients with low-risk scores and high TMB (*p*<0.001; **Figure 5K**). These data suggest that the combination of risk scores and TMB can further enhance the prognostic value for patients.

### 3.5 Association of ferroptosis-hypoxia risk score with clinical characteristics and other classical gastric cancer classification features

**Figure 6:**
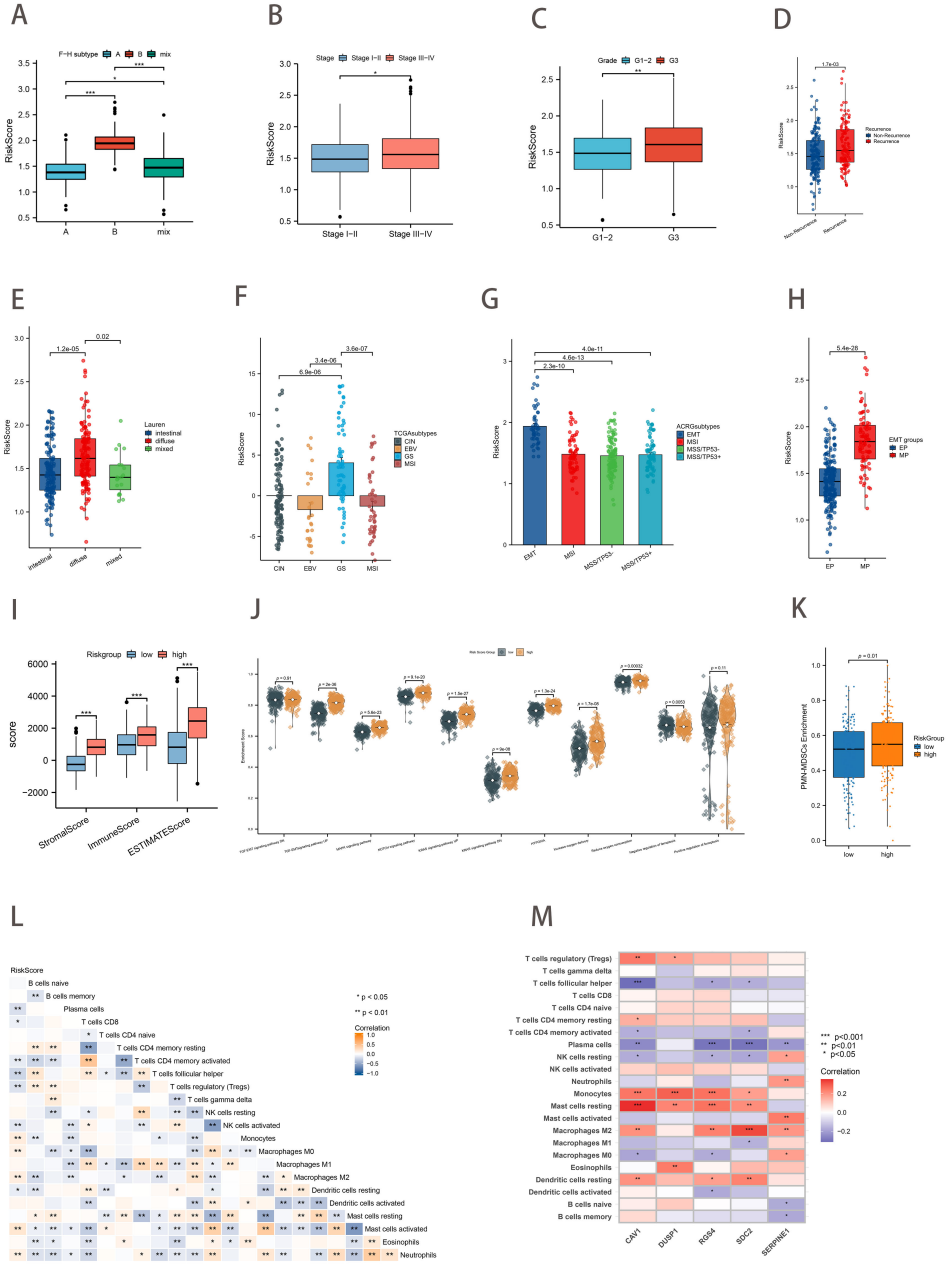
Association of Risk Score with Clinical Characteristics, Other Classification, and Tumor Immune Microenvironment. **(A)** Differences in risk scores between the ferroptosis-hypoxia subtypes **(B–E)** Relationship between risk scores and clinical features such as Stage **(B)**, Grade **(C)**, recurrence **(D)**, and Lauren classification **(E)**. **(F)** Differences in risk scores for TCGA types. **(G)** Differences in risk scores between ACRG types. **(H)** Differences in risk scores for EMT types. **(I)** Differences in ESTIMATE score between risk score groups. **(J)** Differences in biological pathways enrichment between risk score groups. **(K)** Differences in PMN-MDSCs enrichment between risk score groups. **(L)** Heat map of the correlation between risk score and immune cell infiltration; *p<0.05; **p<0.01. **(M)** Heat map of the correlation between five hub genes and immune cell infiltration t; *p<0.05; **p<0.01; ***p<0.001.

We proceeded to investigate the correlation between gastric cancer risk scores and clinical characteristics. The Kruskal-Wallis test demonstrated that risk scores exhibited a statistically significant difference between the F-H subtypes (*p*<0.05; **Figure 6A**). The results were consistent with the prediction, with F-H subtype B exhibiting the highest median risk score and the most unfavorable prognosis, whereas the opposite was true for the A subtype. Patients with a more favorable prognosis exhibited recognized clinical characteristics, including stage I-II and grade 1-2. These patients exhibited relatively low-risk scores (**Figures 6B, C**). Furthermore, risk scores were found to be significantly higher in patients with recurrent gastric cancer (**Figure 6D**). Patients in the diffuse category of the Lauren pathology classification exhibited relatively high-risk scores (**Figure 6E**). Subsequently, the relationship between other molecular features used to classify GC and the risk scores was examined. The TCGA study categorized primary gastric cancer into four distinct subtypes: Epstein-Barr virus (EBV) infection, microsatellite instability (MSI), chromosomal instability (CIN), and genomic stability (GS). In our analysis, patients with the GS type exhibited the highest risk score, while those with the EBV type exhibited the lowest risk score, in accordance with our anticipated results (**Figure 6F**). **Figure 6G** demonstrated that risk scores exhibited a statistically significant difference between EMT and other ACRG subtypes (*p*<0.001). Similarly, the MP subtype exhibited a markedly elevated risk score compared to the EP subtype (*p*<0.001; **Figure 6H**).

### 3.6 Tumor immune microenvironment associated with the ferroptosis-hypoxia risk score

The immune score, stromal score, and ESTIMATE score of the GC samples were calculated using the ESTIMATE algorithm to facilitate the assessment of the immune and stromal components of the TME. Higher ESTIMATE scores represent lower tumor purity, which means higher tumor progression and worse prognosis. The high-risk scoring group exhibited notably elevated levels of the stromal score, immune score, and ESTIMATE score (**Figure 6I**). As anticipated, subsequent ssGSEA analysis revealed that the high-risk score group was associated with stromal activation-related signaling pathways and hypoxia-associated pathways (TGF-EMT, MAPK, NOTCH, Hallmark hypoxia, and HIF-1). In contrast, the low-risk group was enriched in the negative regulation of ferroptosis (**Figure 6J**). Pathologically activated neutrophils (PMNs), called myeloid-derived suppressor cells (PMN-MDSCs), are major negative regulators of anti-tumor immunity. **Figure 6K** showed the significant enrichment of PMN-MDSCs in high-risk group samples (*p*=0.01). Subsequently, we conducted correlation analyses between the risk score and immune cell infiltration (**Figure 6L**). The risk score was found to be positively correlated with macrophage M2 infiltration and negatively correlated with T cells follicular helper, macrophages M1 infiltration. Moreover, the expression of the five key genes was also positively correlated with many immunogenic genes, especially macrophage M2 (**Figure 6M**). These data suggested that the ferroptosis-hypoxia risk score may influence tumor growth and progression by regulating immune cells and matrix activation within the tumor microenvironment.

### 3.7 Therapeutic strategies based on ferroptosis-hypoxia risk scores

**Figure 7:**
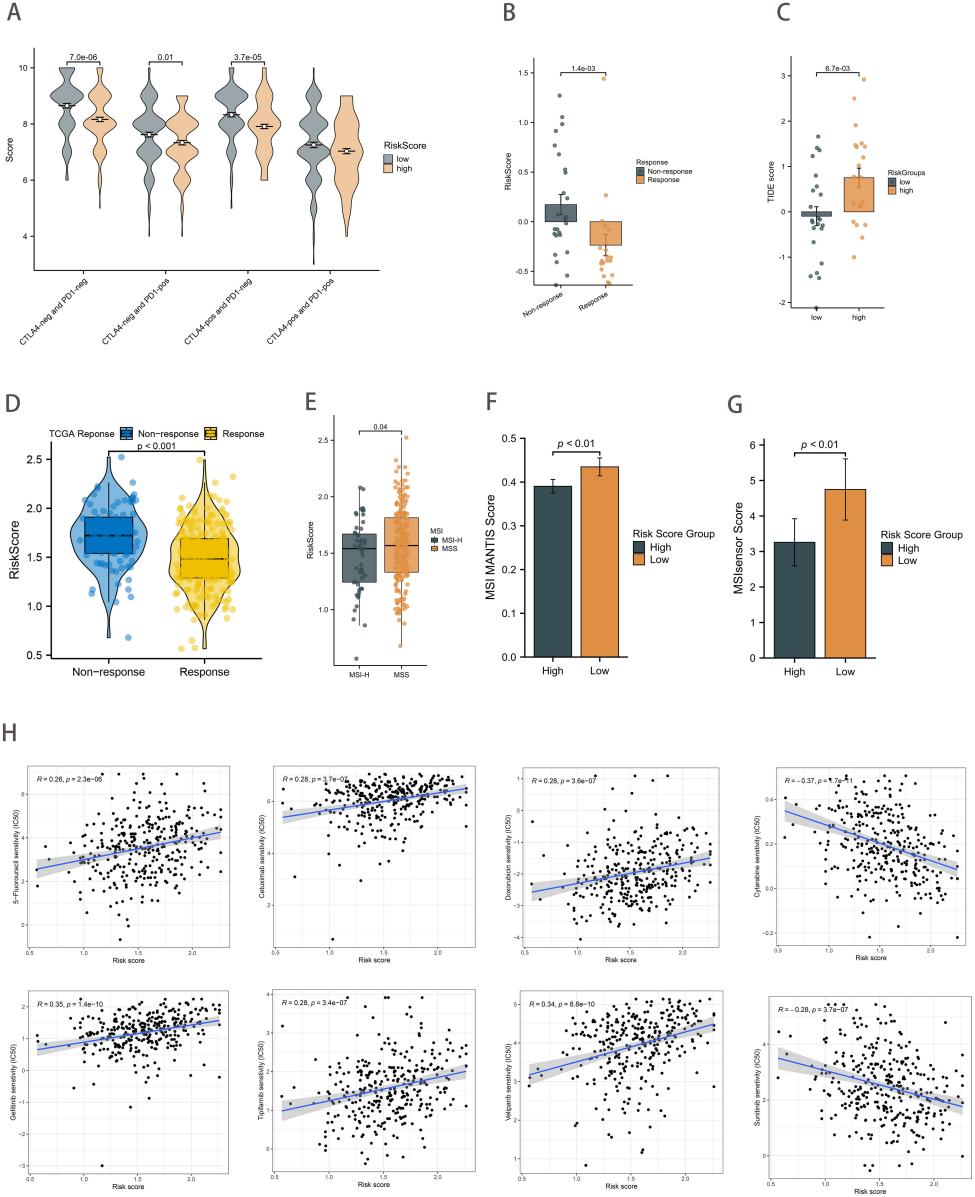
Therapeutic strategies based on risk scores. **(A)** Boxplot showing differences in IPS scores between risk score groups. **(B)** Differences in risk scores between the ICB treatment response and non-response groups in the PRJEB25780 cohort. **(C)** Differences in TIDE scores between the risk score groups from the PRJEB25780 cohort. **(D)** Differences in risk scores between the ICB treatment response and non-response groups from the TCGA cohort. **(E)** Differences in risk scores between MSI states. **(F, G)** Differences in risk score groups between MSI MANTIS score **(F)** and MSIsensor score **(G)**. **(H)** Correlation of risk scores with chemotherapeutic drug sensitivity.

The IPS immunotherapy prediction analysis demonstrated that the low-risk group exhibited favorable therapeutic outcomes in both anti-CTLA-4 and anti-PD-1 immune checkpoint treatment (*p*<0.01; **Figure 7A**). Considering the pivotal role of immunotherapy in cancer treatment, we further investigated the relationship between the risk score and ICBs response by using clinical data from TCGA-STAD and PRJEB25780 (a pembrolizumab-treated clinical trial cohort with metastatic gastric cancer). The risk score in the ICBs treatment response group was demonstrably lower than that in the non-response group (*p*<0.01; **Figure 7B**). Moreover, the TIDE algorithm assessed patients in the PRJEB25780 cohort, and it was found that the risk score was positively correlated with the TIDE score (*p <*0.01; **Figure 7C**). This finding was also validated in the TCGA-STAD cohort (*p*<0.01; **Figure 7D**).

A review of the clinical data on GC patients revealed that those with MSI-H had a lower risk score (*p*=0.04; **Figure 7E**). The MSI MANTIS score is positively correlated with MSI-H status probability (36, 37). By utilizing standard tumor-normal paired sequencing data, the MSI Sensor provides accurate MSI status determination (38). As anticipated, the MSI scores were found to be higher in the low-risk score group (*p*<0.01; **Figures 7F, G**). In conclusion, the evidence presented collectively provides strong support for the predictive efficacy of the risk score in relation to immunotherapy outcomes.

Furthermore, we sought to ascertain the relationship between the IC_50_ of chemotherapeutic agents and risk scores. The findings revealed a significant positive correlation between the IC_50_ of several agents, including 5-fluorouracil, cetuximab, doxorubicin, gefitinib, tipifarnib, and veliparib, and the risk scores. Conversely, the IC_50_ of Cytarabine and Sunitinib demonstrated a strong inverse relationship with the risk score (**Figure 7H**).

### 3.8 Pan-cancer analysis

**Figure 8:**
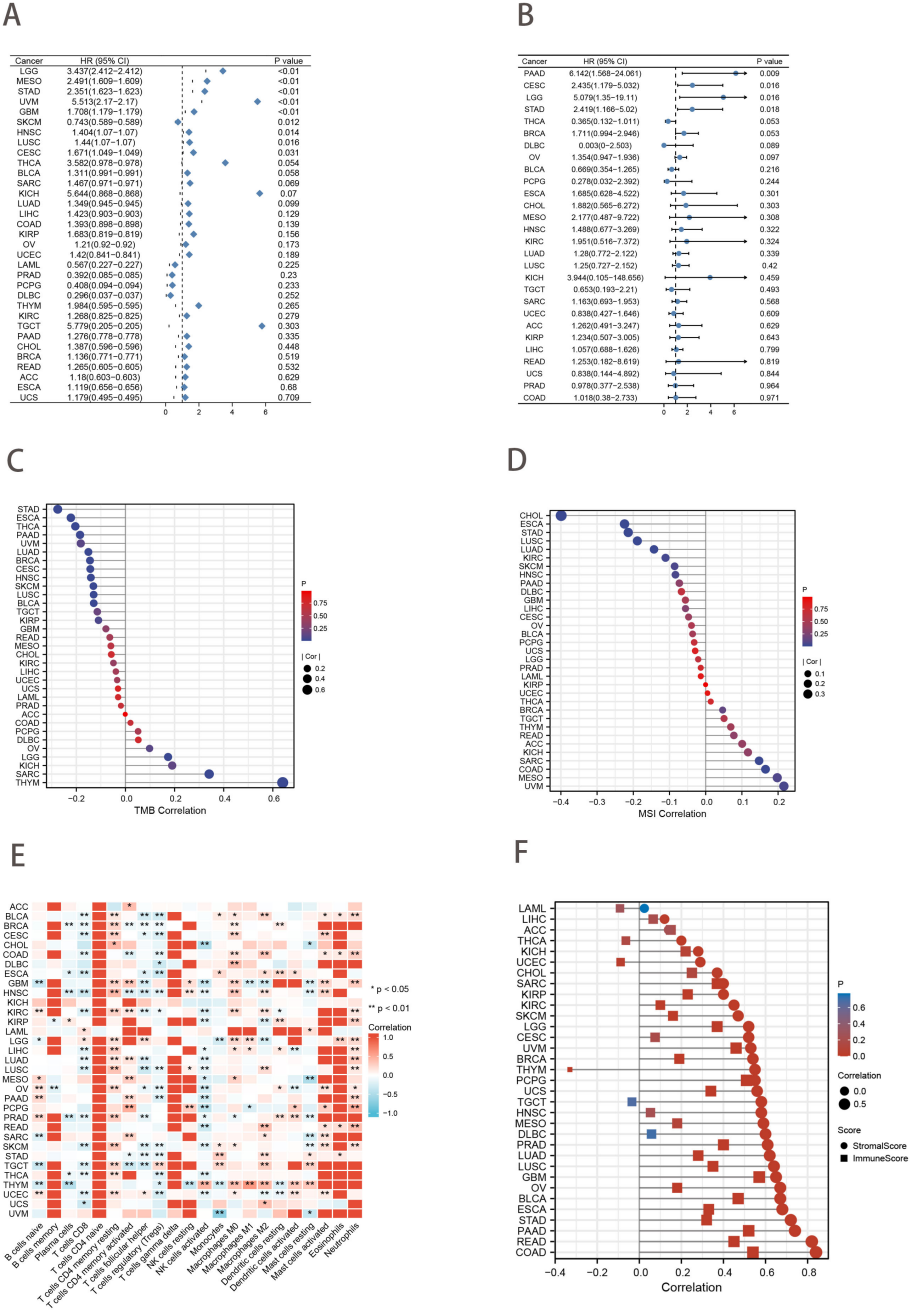
Pan-cancer analysis. **(A, B)** Univariate Cox analysis of risk score when the clinical outcome was OS **(A)** and DFS **(B)**. **(C, D)** Lollipop charts of the Spearman’s Rank Correlation between risk score and TMB **(C)** and MSI **(D)**. **(E)** Pan-cancer landscape associated with risk score and immune cell infiltration. *p<0.05; ***p<0.01. **(F)** Lollipop charts describe the correlation of the risk score in pan-cancer with the ESTIMATE score.

Forest plots from univariate Cox analysis demonstrated that when the clinical outcome was OS, risk scores were predictive of survival for nine cancer types (*p*<0.05; **Figure 8A**). However, when the clinical outcome was disease-free survival (DFS), the risk score was only predictive of 4 cancer types (*p*<0.05; **Figure 8B**). In addition, Spearman rank correlation analysis between risk score and tumor mutational load (TMB) for 33 cancers showed that risk score was positively correlated with TMB in 3 cancers and negatively correlated with TMB in 11 cancers (*p*<0.05; **Figure 8C**). The correlation between risk score and MSI was positive in 2 cancers and negative in 6 cancers (*p*<0.05; **Figure 8D**). We found that risk scores were associated with immune cells in the majority of cancer types (**Figure 8E**). Additionally, ESTIMATE analysis revealed a strong correlation between risk scores and stromal and immune scores (**Figure 8F**).

### 3.9 Validation of ferroptosis-hypoxia related genes expression using scRNA-seq data

The study collected 402 cells from 6 GC samples sourced from GSE112302. After undergoing quality control and normalization, two cells that did not meet the required standards were removed from the study. There was no observed link between the depth of sequencing and the sequences of mitochondrial genes. However, sequencing depth showed a significant positive correlation with total intracellular sequences (R=0.38, **Supplementary Figure 3A**). Analysis of 16,288 genes revealed that 1,500 had substantial intercellular variation and 14,788 had low variation (**Supplementary Figure 3B**).

**Figure 9:**
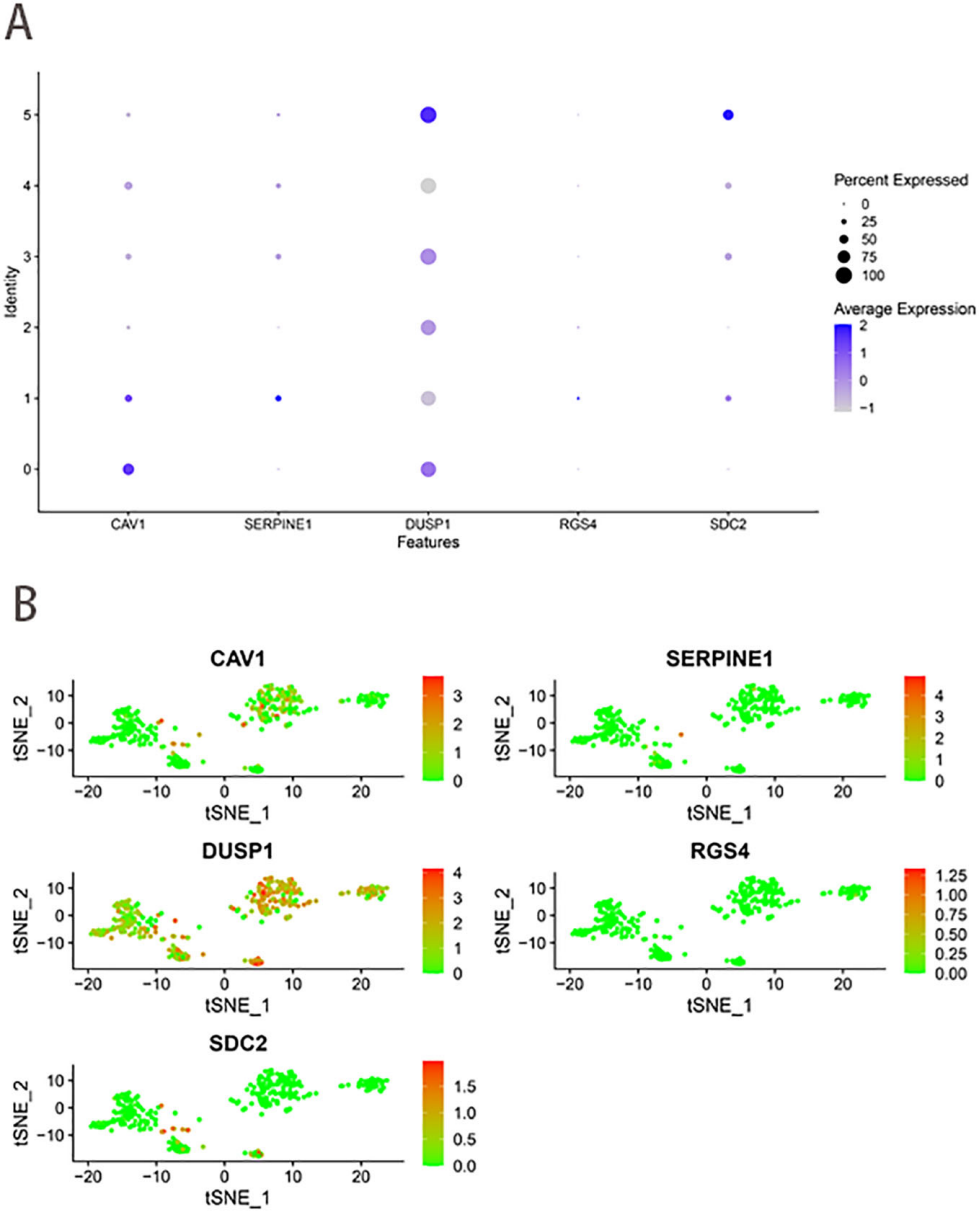
scRNA-seq data analysis. **(A)** The expression levels of five ferroptosis-hypoxia-related genes across the 6 clusters. **(B)** The t-SNE diagrams display expression levels of ferroptosis-hypoxia-related genes.

PCA downscaling results showed no significant separation between GC cells (**Supplementary Figure 3C**). The top 15 principal components (PCs) with substantial distinctions were chosen for further research (**Supplementary Figure 3D**). The 400 GC cells were clustered into 6 clusters according to the tSNE algorithm (**Supplementary Figure 3E**). The 6 clusters were categorized based on marker genes. Clusters 0, 1, 2, 3, and 4 consisted of cancer cells, whereas cluster 5 was linked to macrophages (**Supplementary Figure 3F**). **Figures 9A, B** illustrated the expression levels of five ferroptosis-hypoxia-related genes across the 6 clusters. CAV1 increased in cluster 0, while SERPINE1 and RGS4 demonstrated an increase in cluster 1. Conversely, DUSP1 and SDC2 exhibited an increase in cluster 5.

### 3.10 Clinical cohort verification

**Figure 10:**
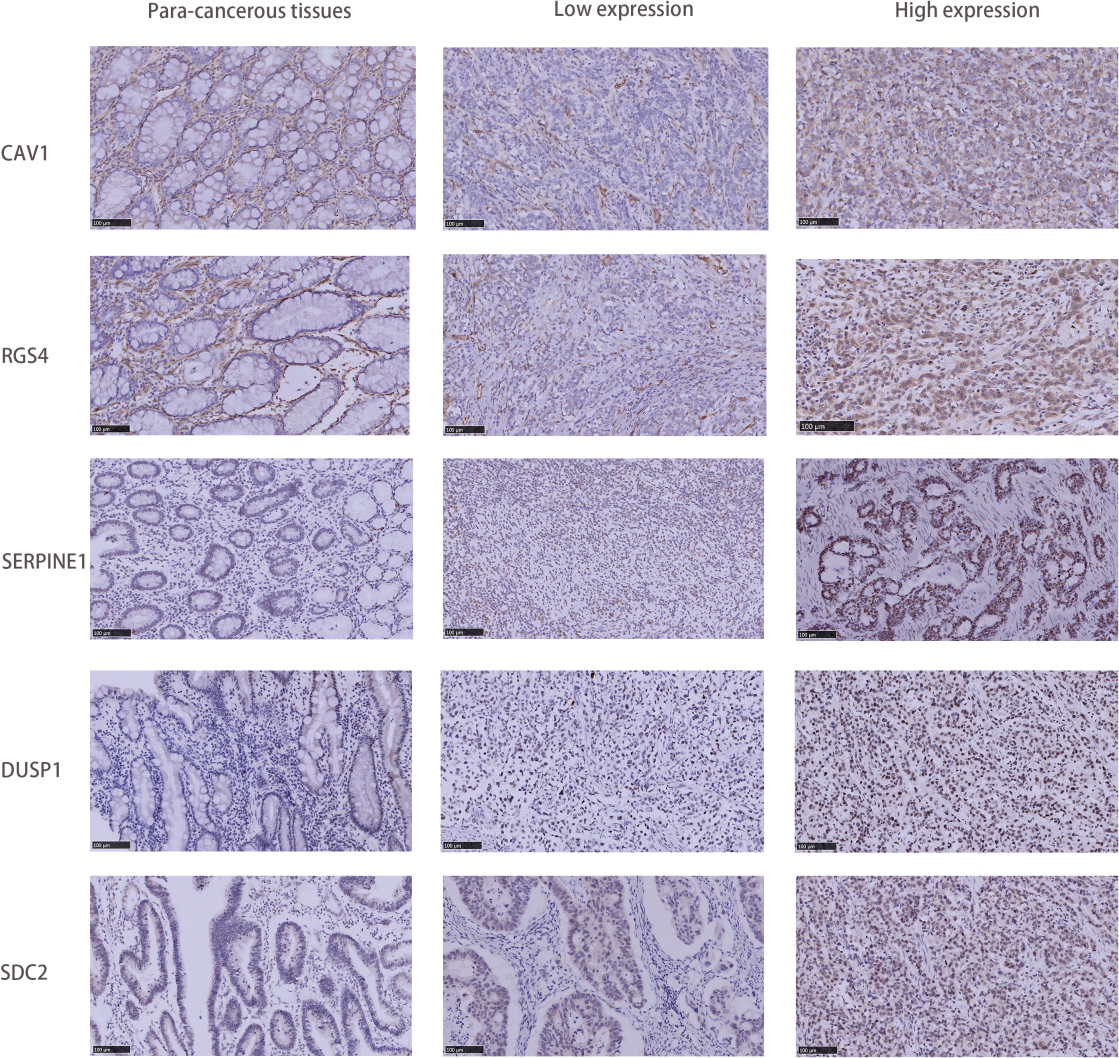
Comparison of SDC2, RGS4, SERPINE1, DUSP1, and CAV1 IHC expression in GC tissues and adjacent tissues.

An immunohistochemical (IHC) investigation assessed the expression levels of hub genes (SDC2, RGS4, SERPINE1, DUSP1, and CAV1) in gastric cancer. The majority of specimens from the validation cohort at our hospital exhibited positive expression of SDC2, RGS4, SERPINE1, DUSP1, and CAV1. Among the aforementioned genes, CAV1 was strongly stained in 13 (43.3%) specimens, SDC2 in 25 (83.3%) specimens, SERPINE1 in 27 (90.0%) specimens, DUSP1 in 28 (93.3%) specimens, and RGS4 in 23 (76.7%) specimens (**Figure 10**). Furthermore, IHC results of candidate genes expression in gastric cancer tissues were found in the HPA database (**Supplementary Figure 4**).

## 4 Discussion

During the development of gastric cancer, metabolic reprograming, genomic instability and differences in the tumor microenvironment can result in the formation of cell clones with entirely different biological behaviors. These cell subsets exhibit notable heterogeneity in proliferation rate, invasion, and metastasis ability and also display varying sensitivities to drugs. The advancement of molecular analysis reveals that even under the same histological diagnosis, there are different genomic changes among patients (39). In recent years, with the advancement of immunotherapy and the identification of novel targets in gastric cancer, considerable progress has been made in treating gastric cancer (40, 41). With the diversification of systemic treatment options for advanced gastric cancer, the accurate selection of composite target inhibitors and personalized immunotherapy regimens has become a research priority. In the future, the exploration direction of advanced gastric cancer is to subclassify patients, identify personalized and efficient whole-course treatment strategies based on molecular typing, and accurately identify the population that may profit from immunotherapy to enhance long-term survival.

In this study, we initially identified genes related to ferroptosis and hypoxia that exhibited differential expression between gastric cancer and normal tissues. The identified DEGs were enriched in immune activation, p53 signaling pathway, HIF-1, angiogenesis regulation, extracellular matrix component regulation, and various tumor-related pathways. By unsupervised cluster analysis of these DEGs, we classified gastric cancer patients into ferroptosis and hypoxia subtypes that differed significantly in prognostic and biological characteristics. The preceding analysis indicates that both the ferroptosis and hypoxia molecular subtypes exhibit favorable prognostic value and possess distinctive tumor-related biological characteristics. Given the close association between ferroptosis and hypoxia, we proceeded to combine the above ferroptosis and hypoxia status into a two-dimensional index classifying patients into three ferroptosis-hypoxia (F-H) subtypes. Our findings indicate that multiple oncogenic classical pathways are activated in the F-H subtype B. Furthermore, F-H subtypes B have increased matrix activity and more notable interstitial features (MP). This indicates that the F-H subtype B may be closely related to the EMT. These mechanisms are thought to suppress the activity of immune cells, and subsequent analysis of immune infiltration also verified the enrichment of immunosuppressive cells in subtype B. In addition, F-H subtype A exhibited increased immune activation, including elevated concentrations of CD8+ T cells, macrophages M1, and CD4+ T cells. The negative regulation of ferroptosis and downregulation of the TGF-EMT signaling pathway were highly expressed in the F-H subtype A. This suggests that two distinct ferroptosis-hypoxia subtypes may profoundly affect the biological behavior and immune microenvironment of GC.

Two gene clusters were identified based on the characteristic DEGs associated with the ferroptosis-hypoxia subtypes. Our findings indicate that the genomic pattern largely correlates with the molecular pattern associated with ferroptosis and hypoxia. Consequently, an extensive evaluation of the molecular characteristics of ferroptosis-hypoxia is crucial for gaining a deep comprehension of GC. Given the heterogeneity of GC, we employed Lasso-Cox regression analysis to identify five characteristic genes based on the expression profiles of the aforementioned genes. This analysis established a prognostic model and risk score, which were subsequently used to divide GC patients into high- and low-risk groups. There was a notable difference in OS between patients in the high-risk group and those in the low-risk group, suggesting that risk score demonstrated favorable predictive efficacy in prognosticating the outcomes of patients with gastric cancer.

As a crucial indicator of ICB efficacy, TMB represents the immunogenicity of the tumor itself. In patients with gastric cancer, higher TMB is associated with improved survival (42). The low-risk group exhibited relatively higher TMB, which is consistent with the results of our study. Furthermore, the correlation between risk scores and clinical features was investigated. Patients with a more favorable prognosis tend to exhibit recognized clinical features, including stage I-II, grade 1-2, and non-recurrent. These patients tend to have relatively low-risk scores. Combined with several previous large-scale classical molecular typing studies of gastric cancer, we found that higher risk scores were significantly associated with diffuse of the Lauren category, GS subtype, EMT subtype, and MP subtype. All of these subtypes represent poorer prognosis and aggressive biological behaviors. The correlation between risk score and EMT phenotype also indicates that a higher risk score may be indicative of stromal activation. In addition, the high-risk score group was associated with lower tumor purity, matrix activation-related signaling pathways, hypoxia-related pathways, and abundant infiltration of M2 macrophages and PMN-MDSCs. Kim et al. proposed that hypoxia-mediated ferroptosis in tumor PMN-MDSCs is a unique targeted immunosuppressive mechanism in the tumor microenvironment. Inhibition of ferroptosis by using genes and drugs can eliminate the inhibitory activity of PMN-MDSCs, slow down tumor progression, and synergize with immune checkpoint blocking to increase the sensitivity of immunotherapy, thereby inhibiting tumor growth (43). The data manifest that the ferroptosis-hypoxia risk score may influence tumor growth and progression by modulating immune cell and matrix activation in the tumor microenvironment. It is, therefore, postulated that risk scores may be helpful for the prediction of immunotherapy. Therefore, we subsequently performed immunotherapy prediction analyses by multiple routes.

Pabolizumab has received approval for treating solid cancers characterized by high microsatellite instability (MSI-H) or mismatch repair defects (dMMR), making it the first ICBs to receive full approval as “pan-cancer” treatments (44, 45). A higher response rate to ICBs has been observed in patients with MSI-high (MSI-H) tumors compared with patients with microsatellite instability low (MSI-L) cancers (46). Among the numerous molecular markers for predicting the efficacy of immunotherapy, the clinical value of MSI has been consistently demonstrated in various clinical studies of gastric cancer (47). A review of the clinical data of GC patients revealed that MSI-H patients exhibited a lower risk score. Furthermore, the low-risk group exhibited higher MSI MANTIS and MSIsensor scores. The IPS analysis demonstrated that the low-risk group exhibited a favorable therapeutic response to both anti-CTLA-4 and anti-PD-1 immune checkpoint therapy. The predictive role of risk scores on immunotherapy response was validated in the TCGA-STAD and PRJEB25780 cohorts. The risk score of the ICB treatment response group was found to be significantly lower than that of the non-response group using multiple methods.

The results of the drug sensitivity analysis indicated a clear positive correlation between the IC_50_ and the risk score of various gastric cancer treatments, including 5-fluorouracil, cetuximab, doxorubicin, and gefitinib. One potential avenue for future research is the stratification of patients based on risk-scoring systems, the screening of immunotherapy-sensitive patients, and the identification of novel strategies to overcome chemotherapy resistance. These endeavors could provide invaluable insights for the advancement of more efficacious treatment modalities. Moreover, the risk score was extended to a pan-cancer analysis. The risk score exhibited varying degrees of sensitivity across different cancers. This provides a foundation for further research.

Finally, the expression of the five critical genes identified through the screening process was verified. Batch RNA sequencing (RNA-Seq) techniques provide transcriptional profiles of cell populations or average expression levels of tissues but lack the capacity to identify gene expression patterns in individual cells (48, 49). The advent of single-cell RNA sequencing (scRNA-seq) has enabled researchers to provide a comprehensive characterization of genetic complexity at the cellular level, thereby contributing to a more profound comprehension of cellular heterogeneity (50). Single-cell sequencing analysis revealed that the expression of these five genes was significantly enriched in cancer cells. In addition, DUSP1 and SDC2 exhibited an increase in macrophages in the tumor microenvironment. In clinical gastric cancer (GC) specimens without chemotherapy or targeted therapy, the expression of these five genes was significantly higher in cancerous tissues than in paracancerous tissues.

To improve the reliability and generalization of our study, we utilized gene expression files from 1121 samples across four datasets. We employed the ComBat method to eliminate the batch effect of gene expression data. The accuracy of the dataset is contingent upon the quality and availability of the original data. Additionally, some studies had limitations in the number of marker genes utilized, excluding some meaningful molecular targets in gastric cancer, such as *Claudin18.2*. Consequently, it is imperative to continuously enhance research methods and broaden the scope of research to improve model accuracy and effectiveness. Our current work consists of preliminary validation experiments, and the experimental results require validation in a large multicenter GC cohort. Moreover, further functional and mechanistic studies are necessary to elucidate hypoxic-ferroptosis interactions and potential cancer pathogenesis. Despite these limitations, this study’s results may still offer new treatment strategies for GC. The challenges of chemotherapy resistance and immunotherapy insensitivity in treating gastric cancer are pressing issues. Researchers have been working on developing new therapies based on hypoxic-ferroptosis and have shown promising results in preclinical studies. It is believed that delving into hypoxic-ferroptosis in the tumor immune microenvironment will offer a new treatment strategy for advanced gastric cancer patients.

## 5 Conclusions

This study conducted a comprehensive assessment of the molecular patterns of ferroptosis-hypoxia in GC. To assess the ferroptosis-hypoxia condition of each patient, we have also furthermore developed a risk score. According to the results, risk scores could effectively assess the genetic mutation landscape of cancer, tumor microenvironment, survival prognosis, and immunotherapy response. In light of these findings, we might consider applying the risk score as a basis for categorizing GC. This could help in the development of targeted medicines and designed clinical trials.

## Data Availability

The datasets presented in this study can be found in online repositories. The names of the repository/repositories and accession number(s) can be found in the article/**Supplementary Material**.
