# Supplementary figures and images for: Integrative analysis of ferroptosis in the hypoxic microenvironment of gastric cancer unveils the immune landscape and personalized therapeutic strategies
Source: Front Oncol. 2025 Jan 13;14:1499580. doi: 10.3389/fonc.2024.1499580 (PMC11769819; doi:10.3389/fonc.2024.1499580)

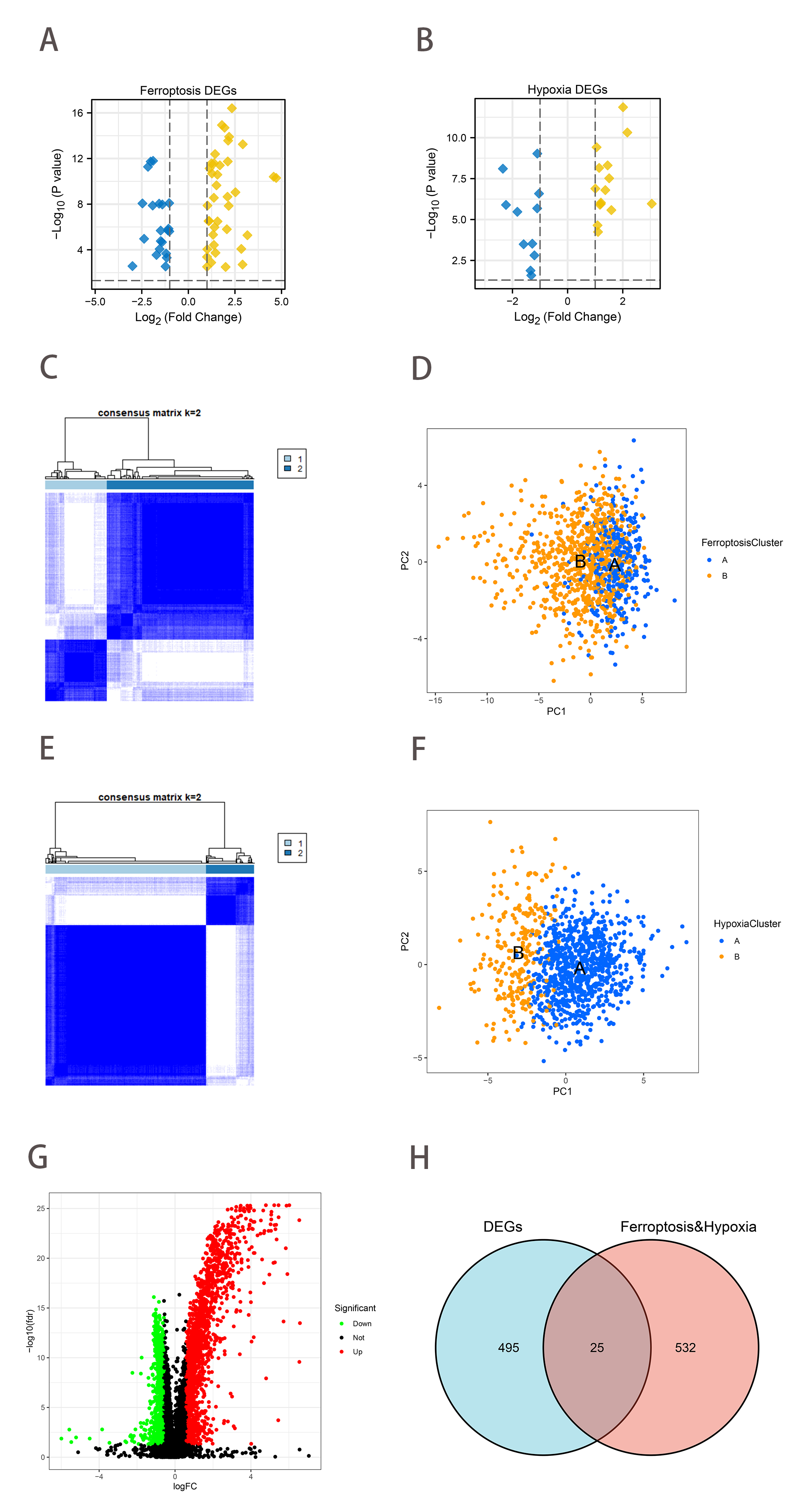

Supplement: Supplementary file 1 [file Image1.tif]

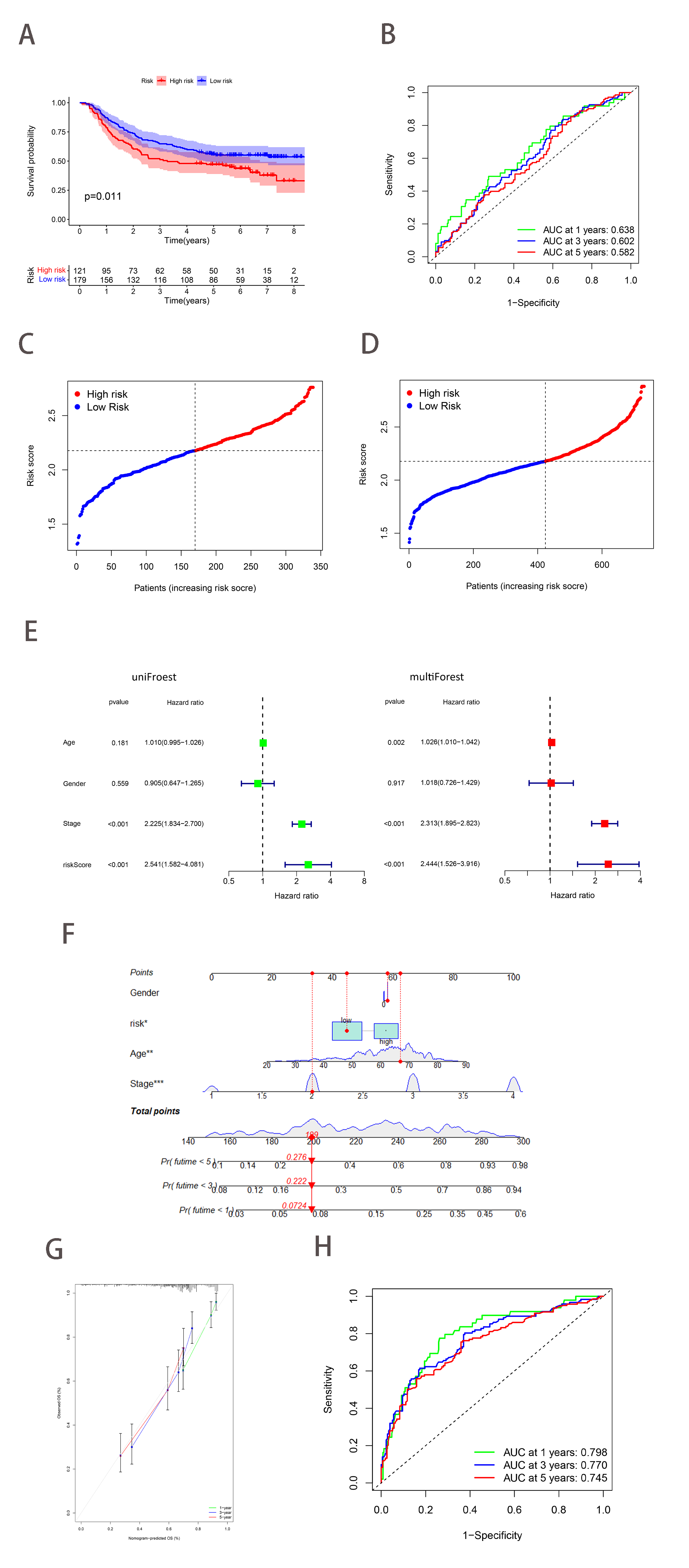

Supplement: Supplementary file 2 [file Image2.tiff]

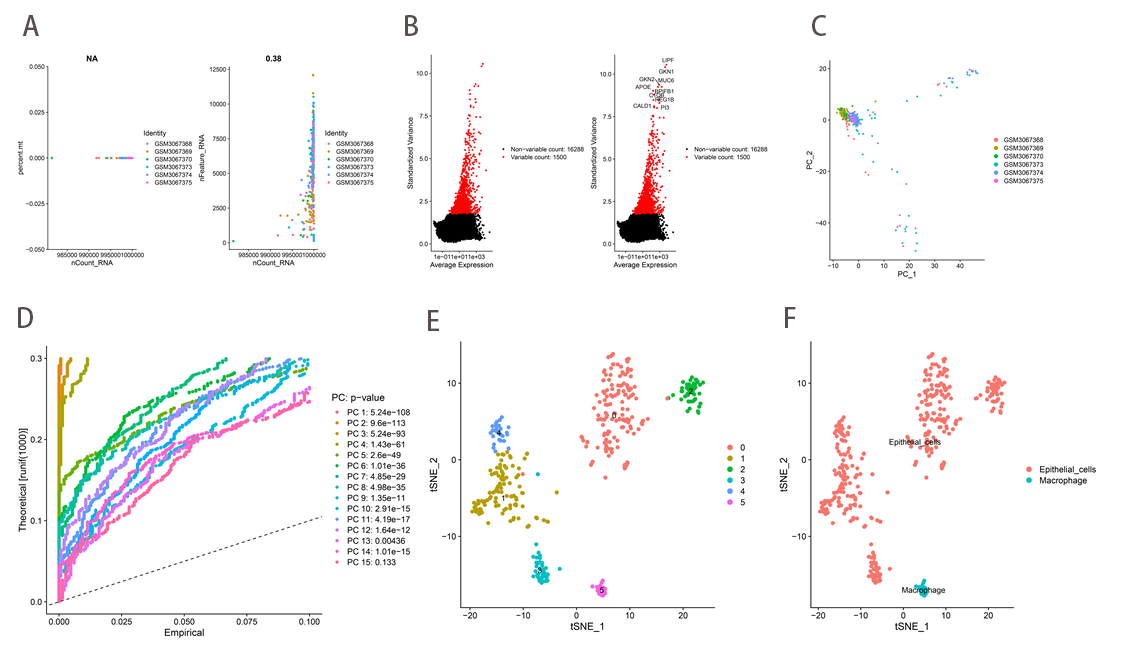

Supplement: Supplementary file 3 [file Image3.tif]

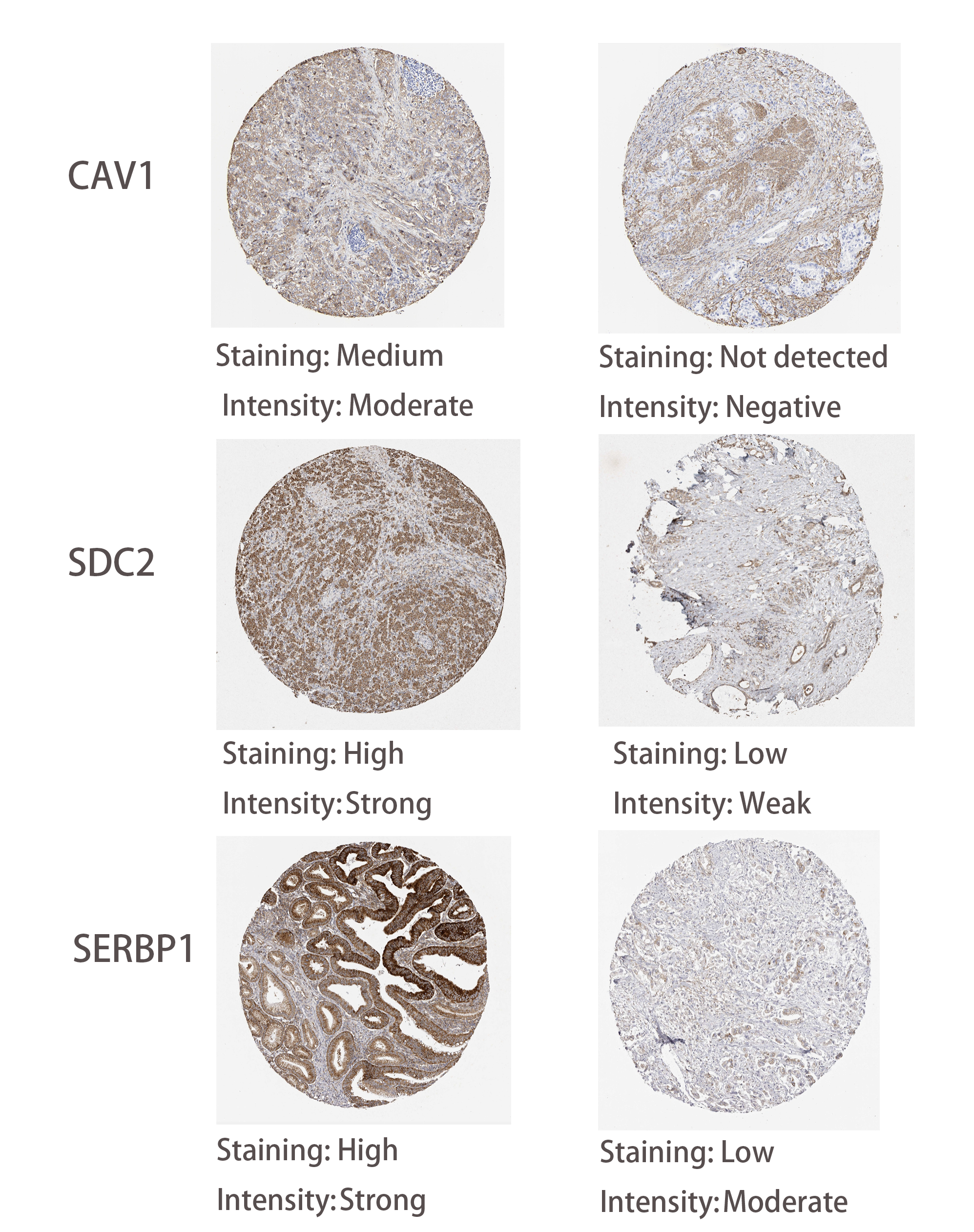

Supplement: Supplementary file 4 [file Image4.tif]
